# Evidence of soft bound behaviour in analogue memristive devices for neuromorphic computing

**DOI:** 10.1038/s41598-018-25376-x

**Published:** 2018-05-08

**Authors:** Jacopo Frascaroli, Stefano Brivio, Erika Covi, Sabina Spiga

**Affiliations:** Laboratorio MDM, IMM-CNR, Via C. Olivetti 2, 20864 Agrate Brianza, (MB) Italy

## Abstract

The development of devices that can modulate their conductance under the application of electrical stimuli constitutes a fundamental step towards the realization of synaptic connectivity in neural networks. Optimization of synaptic functionality requires the understanding of the analogue conductance update under different programming conditions. Moreover, properties of physical devices such as bounded conductance values and state-dependent modulation should be considered as they affect storage capacity and performance of the network. This work provides a study of the conductance dynamics produced by identical pulses as a function of the programming parameters in an HfO_2_ memristive device. The application of a phenomenological model that considers a soft approach to the conductance boundaries allows the identification of different operation regimes and to quantify conductance modulation in the analogue region. Device non-linear switching kinetics is recognized as the physical origin of the transition between different dynamics and motivates the crucial trade-off between degree of analog modulation and memory window. Different kinetics for the processes of conductance increase and decrease account for device programming asymmetry. The identification of programming trade-off together with an evaluation of device variations provide a guideline for the optimization of the analogue programming in view of hardware implementation of neural networks.

## Introduction

Common hardware logic based on von Neumann paradigm is increasingly facing a bottleneck due to data transfer between separated processing and memory units. To overcome this hindrance, new architectures have been proposed mimicking the co-localization of memory and logic that takes place in our brain^1–3^. Specific bio-inspired chips possess the ability of on-line learning, adaptation, inference and classification, which can be exploited in distributed and mobile applications whenever the target of low power operation is met^4,5^. In analogy with biology^6^, neuromorphic implementations rely on neuron units connected through synaptic elements with variable efficacy of coupling, represented by a synaptic weight. Because of the large number of synapses compared to neurons^7,8^, the full potential in terms of scalability, low power and high density can be unlocked only by developing a dedicated device with the ability to dynamically change the synaptic efficacy in response to variable input conditions^9^. In this respect, memristive devices and especially resistance random access memories (RRAM)^10,11^ have been recently identified as ideal candidates^8,12,13^ because of their simple two terminal structure and promising scalability potential^14^, non volatile memory^15,16^ and low power consumption^17^. Examples of network applications including RRAM devices have been demonstrated both in small scale arrays^18,19^ and at simulation level to implement pattern recognition^20–26^ and other classification tasks^27^.

**Figure 1:**
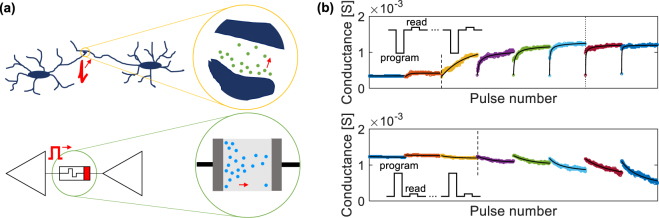
(**a**) Schematic analogy between ionic motion in a biological synapse (e.g. Na^+^, Ca^2+^ ions), in between two neurons, and in an RRAM device (e.g. metal or oxygen ions) connecting two electronic neuron units. (**b**) Examples of conductance series of potentiation (top) and depression (bottom) operations obtained for 300 identical pulses with Δ*t* = 10 *μ*s. Each colour corresponds to a different series of 300 pulses starting from a similar initial state and operated with increasing absolute potential between 0.55 V (left) and 0.9 V (right) with a step of 50 mV.

In RRAM devices, the synaptic weight is mapped as conductance value of the memory element and can be manipulated in response to external voltage stimuli^28^. Despite bistable synapses have been recognized as possible candidates for artificial synapses^21^, to enhance the storage capacity of the network, synaptic devices should preferably exhibit gradual weight change^29^. A gradual conductance change can be obtained in RRAM as a result of the device dynamics, which is governed by the motion of ionic species inside the functional material of the metal-oxide-metal stack^30,31^. Here lies the intriguing analogy between the ionic motion at the base of resistance switching and the motion of the vesicles in the junctions between neurons characteristic of biological synaptic elements, as illustrated in Fig. 1a.

The optimization of artificial synaptic devices for neuromorphic applications remains an open issue. Increasing the degree of analogue operation corresponds to reducing the weight modification in response to discrete voltage pulses. However, due to device variations, it is usually not straightforward to consider deterministically separated conductance levels and the multi-level capability should be considered in a probabilistic fashion^19,32^. Moreover, for specific synaptic applications, some functional non-idealities exist in physical devices that could affect the network performance, such as non linear weight update and asymmetric behaviour between the processes of conductance increase (potentiation) and decrease (depression)^33,34^. Several works concentrate on optimizing the material stack to improve the linearity of the conductance change^35,36^. However, the linear behaviour is usually displayed only up to a certain number of pulses and in a restricted memory window. Another class of researches analyzes the impact of device dynamics and number of conductance levels on the performance of simulated neuromorphic networks^34,37,38^. Network simulations give some useful hints on how the details of synaptic plasticity influence the learning process^39^, though only theoretical conductance changes or representative device results are routinely considered. Indeed, operating parameters strongly determine the device dynamics and a complete device characterization in a diverse range of programming conditions is necessary to optimize the analogue behaviour and identify the robustness of the analogue operation.

A major source of deviation from the ideal linear weight update in physical devices is the presence of limits to the maximum conductance span achievable for a given programming condition due to a finite memory window. Conductance saturation close to the boundaries causes a deviation from linearity of the conductance dynamics and introduces a state-dependent conductance update. While Ziegler *et al*.^40^ identified the basic aspects of Hebbian learning in bounded memristive devices, the presence of boundaries to the maximum synaptic strengths imposes a constraint to the storage capability^41^ and impacts the learning process and the decay of the stored memory due to ongoing plasticity^29^. On the other hand, in the most general case of unbalanced probabilities of synaptic potentiation and depression, Fusi and Abbott^42^ noticed the advantage in terms of memory capacity of softly bounded synaptic weights, in which the weight boundaries are approached asymptotically as the weight update tends to zero.

In this work, we study with a systematic approach the gradual evolution of conductance dynamics of filamentary HfO_2_-based resistive RAM upon train of identical programming pulses and as a function of a wide range of pulse voltage and time width parameters. We show that the soft bound phenomenological model identified by Fusi and Abbot^42^, however simple, captures the salient features of the experimental conductance change related to a state-dependent update and to a soft approach to the conductance boundary values. The model is then applied as metrics to identify the degree of analogue operation in the different programming regimes, namely no switching, gradual conductance change stimulated by train of identical pulses and conductance change achieved by a single programming pulse (binary regime). Further, we discuss the relationship between the experimental results, the proposed model and the device physics. Device non-linear switching kinetics is recognized as the origin of the transition between different dynamics and motivates the crucial trade-off between the degree of analogue modulation, programming symmetry of the potentiation/depression operations and memory window, as a general feature of filamentary-type resistance switching devices. Finally, evaluating the variability and reproducibility around the average resistance evolution allows to give a quantitative estimation of device performances in the different regimes of operation. While several publications highlight the relative immunity to device variations of spiking neural networks due to their intrinsic properties^25,37^, the reported analysis provides some insight on the actual variation and robustness of analogue operation.

## Results

### Representative features of conductance dynamics

The analysed devices present a TiN/HfO_2_/Ti/TiN structure and their operation is based on a filamentary switching mechanism, according to previous papers^16,43,44^, initiated by a forming process as described in the Methods section. Conductance increase (potentiation) and decrease (depression) dynamics is characterized by pulsed measurements in a wide space of the programming parameters varying the time width and voltage amplitude (Δ*t* and Δ*V*) of the applied pulses while keeping constant the initial state of the memory cell. The aim is to identify the operating regions for increasing and decreasing connectivity strength as a function of the programming conditions of the incoming pulses.

Trains of identical pulses were applied to the device and its conductance was continuously monitored by a reading operation after each pulse. This procedure was repeated after reinitializing the device to a similar initial state and varying the pulse voltage or time width for each series. Further details can be found in the Methods Section. An example of the conductance sequences resulting from 300 identical pulses repeated with increasing Δ*V* and fixed Δ*t* = 10 *μ*s can be found in Fig. 1b for both potentiation and depression operations. The two reported examples already anticipate some of the main experimental findings. For low enough Δ*V*, *i*.*e*. in the first few sequences up to the dashed line, no switching occurs and the conductance value does not change during the pulse sequence since the effect of each pulse is so low to result in no visible change of the cumulative conductance. For pulse amplitudes in between the dashed and the dotted lines, the conductance modification produced by each pulse becomes appreciable and sums up to produce a gradual analogue potentiating or depressing trend. Increasing Δ*V* further, the effect of the first pulse becomes gradually dominant and eventually it produces most of the overall conductance change (right side of the dotted line for potentiation), resulting in a digital switching behaviour. This is particularly evident in the last potentiation series of Fig. 1b, for which only two conductance levels can be distinguished. To observe a similar digital behaviour for the depression series, Δ*t* should be further increased as reported in the Supplementary Fig. S1.

In summary, three main switching regimes can be identified as separated by two thresholds roughly identified by dashed and dotted lines in Fig. 1b. The first threshold (in the following referred to as switching threshold) marks the transitions from no switching to the activation of gradual switching, while the second threshold indicates the onset of digital switching. The different position of the switching thresholds for potentiation and depression, though roughly placed in this figure just as a guide for the eye, already remarks the asymmetry in terms of conductance dynamics of filamentary RRAMs. In the following, a complete experimental analysis will allow the quantitative positioning of the switching thresholds in relation with the kinetics of the switching process and the demonstration of the bounded nature of the conductance dynamics.

### Bounded nature of the cumulative conductance change

**Figure 2:**
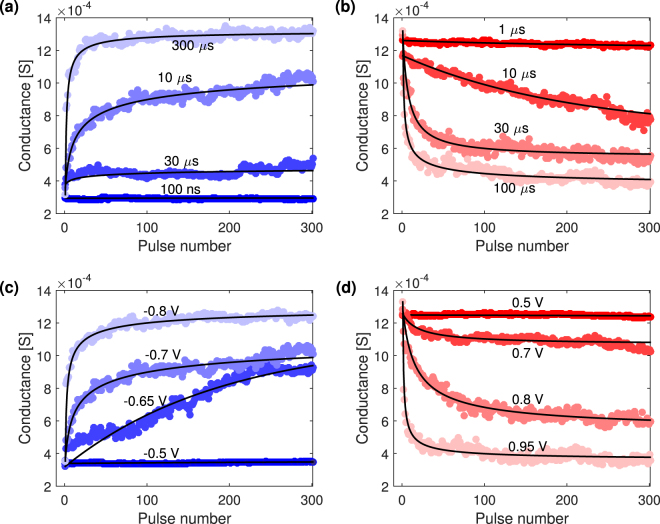
Sequences of 300 identical pulses for different programming conditions. In black lines, fit to data of the generalized soft bound model. (**a**) Potentiation operations for a fixed Δ*V* of −0.7 V at different Δ*t*; (**b**) Depression operations operated at Δ*V* = +0.9 V and different Δ*t*; (**c**) Potentiation curves obtained at Δ*t* = 10 *μ*s and different Δ*V*; (**d**) Depression curves for Δ*t* = 100 *μ*s and different Δ*V*.

Figure 2 shows representative conductance trends produced by sequences of 300 identical pulses. In each frame one programming condition, namely Δ*V* or Δ*t*, has been fixed letting the other parameter vary. The conductance dynamics evolves among the three regions of no substantial modification, gradual switching and abrupt switching (from dark to clear colour tones) increasing either the time width at constant voltage (Fig. 2a,b) or the voltage amplitude at constant time width of the pulses (Fig. 2c,d). A complete set of plots containing all the acquired curves can be found in the Supplementary Fig. S2.

A significant feature common to all the conductance curves is the attainment of conductance saturation for a sufficiently high number of pulses. In particular, even when curves look almost linear for a limited pulse number, increasing the number of pulses will eventually drive the device in saturation and the overall trend will flatten and deviate from linearity. The non-linear behaviour is linked to the uneven conductance changes induced by each pulse. In particular, the effect produced by a single pulse gets smaller and smaller as the saturation level is asymptotically approached. This suggests that conductance modulation is a function of the current device state.

In Fig. 2 it can also be noticed that increasing the strength of the programming conditions (*i*.*e*. |Δ*V*| or Δ*t*) the conductance window expands accordingly at the expense of the smoothness of the conductance change.

### Features of conductance dynamics

**Figure 3:**
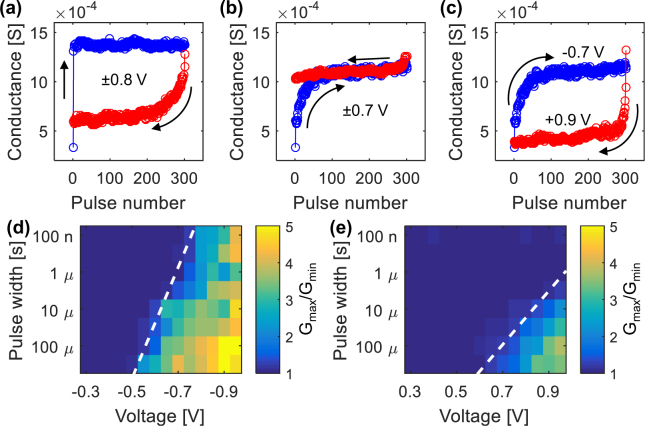
(**a**–**c**) Examples of potentiation (in blue) and depression (in red) operations to evidence the device asymmetry. Δ*t* is fixed at 100 *μ*s in all cases. The absolute Δ*V* is set to 0.8 V in (**a**); 0.7 V in (**b**); 0.7 V for potentiation and 0.9 V for depression in (**c**). (**d**,**e**) Memory windows, defined as the conductance ratio after 300 identical pulses, as a function of Δ*t* and Δ*V* for the potentiation (**d**) and depression (**e**) processes. A dashed line is added to highlight the switching threshold, defined for a minimum variation of 10%.

Figure 3 emphasizes the experimental asymmetries between conductance evolution for potentiation and depression operations, which rise as a consequence of the inherent differences in the corresponding switching kinetics. It is demonstrated that providing the device with the same strength of the stimulus (same Δ*t* and absolute Δ*V*), either qualitatively different smoothness of the conductance change (Fig. 3a) or unmatched conductance windows (Fig. 3b) are obtained comparing potentiation and depression. Analogue modulation for both operations is achieved if an unbalanced Δ*V* is introduced, as in Fig. 3c.

The source of such asymmetries lies in the inherently different switching kinetics (Fig. 3d,e) of the processes responsible for conductance potentiation and depression, *i*.*e*. filament formation and dissolution. The memory window (defined as the conductance ratio G_max_/G_min_ after 300 pulses) is portrayed as a function of the pulse programming parameters. The memory window is close to 1 for low Δ*V* and Δ*t*, meaning that no switching occurs, while increasing both programming parameters a switching threshold is crossed (dashed line in Fig. 3d,e) which marks the onset of the conductance change. Above threshold, higher programming values correspond to higher memory windows. The switching threshold is arbitrary considered as the minimum voltage (or time width) that has to be applied to produce a 10% conductance change and we will demonstrate in the following that it corresponds to the threshold for gradual conductance change. The logarithm of the switching time follows a straight line implying an exponential relation between Δ*V* and Δ*t*^32^. Such exponential dependence has been referred to as an extension of the voltage – time dilemma^45^ and descends from the non-linear switching kinetics typical of valence change memory cells, as illustrated in ref.^46^. Different slopes of the lines identifying the switching kinetics can be seen in potentiation and depression, in general agreement with results in the literature^46–49^. In fact, the voltage – time slope of 50 mV/decade evaluated from Fig. 3d for the potentiation process corresponds to the one reported for devices with similar composition^46^. On the other hand, the steeper slope of 130 mV/decade estimated from Fig. 3e highlights the slower switching kinetics of the depression process^47,48^. This finding evidences that an inherent asymmetry of the switching mechanisms associated with potentiation and depression operations exists and is reflected into the conductance dynamics. For instance, it is immediately evident that the main effect of the slower kinetics of depression results in an overall lower memory window when compared to potentiation for the same applied absolute values of the voltage. A quantitative assessment of the characteristic features reported in the latter two paragraphs will be provided in the following.

### Analysis of the conductance update

In the previous section, several characteristics of the device conductance dynamics have been introduced. The main features to take into consideration when modeling the device are the presence of limits to the cumulative conductance modulation and a non-constant conductance change that progressively decreases as the conductance boundaries are approached. Moreover different device dynamics, which can be divided into three main switching regimes, can be observed. Therefore, a device model should correctly reproduce all these salient features. A general formulation of a synaptic device displaying maximum and minimum weight boundaries approached with smaller and smaller steps was proposed by Fusi and Abbott^42^ as a multiplicative update rule with a weight-dependent weight update. When the synaptic weight is comprised for simplicity between 0 and 1, the following generalized soft bound equations (1) and (2) were proposed for the incremental (*δw*_+_(*w*)) and decremental (*δw*_−_(*w*)) weight change in potentiating and depressing events, respectively.

1 $$\delta {w}_{+}(w)=\alpha {(1-w)}^{\gamma }$$

2 $$\delta {w}_{-}(w)=-\,\alpha {w}^{\gamma }$$

In this model *α* is a multiplicative parameter which determines the magnitude of modification induced on the synaptic strength by a plasticity event, thus 1/*α* is proportional to the number of discrete accessible synaptic states. To add more generality to the update rule, a second positive parameter *γ* is added at the exponent of the recursive sequence, adapting the dependency of the weight update on the synaptic state. It is worth mentioning that a constant weight change, resulting in a linear weight dynamics, is described by a value *γ* = 0. A first formulation of weight dependent update rule is the one neglecting the parameter *γ* (or equivalently *γ* = 1). Letting the parameter *γ* varying above unity constitutes a general formulation of a weight dependent update rule. The description of the influence of *α* and *γ* parameters on the weight dynamics is reported in the Supplementary Fig. S3.

In the following, we show that this simple synaptic model can be applied to a physical HfO_2_-based device and is able to capture the conductance dynamics in a complete set of regimes of operation, from no switching, to gradual analogue switching, to digital deterministic switching.

**Figure 4:**
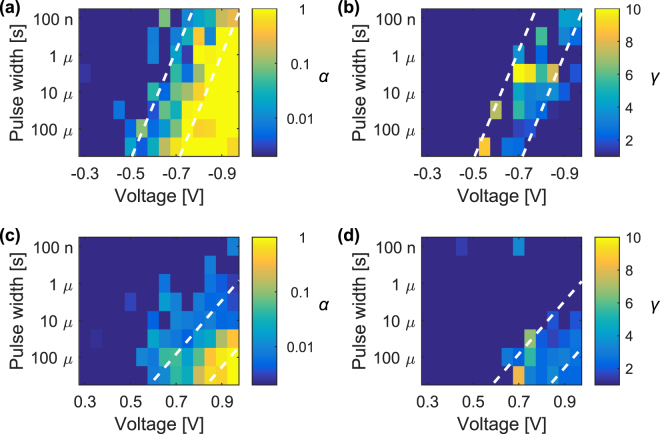
*α* and *γ* parameters extracted from fit to data of a generalized soft bound law as a function of Δ*t* and Δ*V*. (**a**,**b**) Refer to potentiation operations, while (**c**,**d**) refer to depression. In each figure, the dashed lines serve as visual distinction of the three switching regions: no switching (left), analogue (centre) and digital (right) behaviour.

The experimental conductance curves previously discussed are fitted according to the generalized soft bound model (further details are reported in the experimental section) and the fitting lines are visible in Figs 1b and 2 (see also Supplementary Fig. S2) as black solid lines superposed to the measured data points. The resulting fitting parameters are mapped into the pulse time width and voltage space in Fig. 4a–d for potentiation and depression, respectively. For low voltage and short pulses (top left corner of the maps), the pulses produce no appreciable conductance variation and the flat response can be well reproduced by *α* ≈ 0, since only one stable effective state exists in the system. At the opposite, for high values of Δ*V* or Δ*t* where the device exhibits a digital behaviour, the system has only two stable states since one single pulse is sufficient to produce the maximum conductance change and the conductance trend can be reproduced by *α* ≈ 1. In between these two extremes, the device shows a gradual variation of the conductance before reaching saturation, and *α* assumes values 0 < *α* < 1. Two dashed lines have been inserted in the 2D maps to highlight the separation between the three switching regions. The leftmost lines correspond to the switching thresholds extracted from Fig. 3d,e and separate the region of no significant conductance variation (no switching region) from the analogue switching region, while the rightmost lines are positioned at *α* values close to 1 and signal the onset of the digital switching region.

It should be stressed that a fit to a simple soft bound model (*γ* = 1) is roughly able to replicate the different regimes. This would be equivalent to fit the cumulative conductance with an exponential law. However, in particular for intermediate states slightly above the switching threshold where the device shows a richer multi-state behaviour, this can not be accurately reproduced by the simple one-parameter law. To this end, the introduction of the second parameter *γ* intervenes to round off and adjust the dynamics closer to the experimental trend. While the value of *γ* has no real influence when approaching the two extremes of no switching and digital switching and *γ* ≈ 1 can well fit the data, in the region with analogue modulation *γ* assumes values above 1. A summary of the values of *γ* extracted from fit is reported in Fig. 4b,d for potentiation and depression, respectively.

In potentiation, *α* increases rapidly above the switching threshold and eventually comes close to 1 in a large portion of the inspected (Δ*V*, Δ*t*) space (Fig. 4a). The region with accessible analogue behaviour, where the fitting procedure locates the highest number of levels, can be best visualized by the region with *γ* above 1 in Fig. 4b. This region has a width of approximately 200 mV and follows the general slope of 50 mV/dec previously identified for the switching dynamics, so that a careful choice of Δ*V* should be made to achieve analogue programming. Unlike potentiation, during depression the device goes in an almost digital (2 states) behaviour, which is represented by *α* ≈ 1, only in the bottom right corner of Fig. 4c. This happens since in depression the threshold identifying the digital behaviour is shifted to higher pulse programming values than in potentiation.

As discussed above, in the time – voltage plot *α* and *γ* follow the same trend of the memory window. This becomes apparent by comparing Figs 3d,e and 4. Indicatively, the best analogue operation can be achieved slightly above the switching threshold. This leads to a fundamental consideration about the necessary trade-off between multi-level behaviour and window of operation. For the considered device, in the area of analogue switching roughly identified in Fig. 4, the memory window does not exceed a value of 4 in potentiation and 3 in depression, above which digital switching prevails.

From general considerations based on mean-field simulations, Fusi and Abbott^42^ demonstrate that in a learning network, in the general case of unbalanced rate of potentiation and depression events, hard weight boundaries (*e*.*g*. linear weight update and a hard cut-off at the boundaries) result in a sub-linear relation between storage capacity and number of weight levels (1/*α*). Conversely, softly bounded synapses with *γ* > 0 ensure the memory capacity to always scale linearly with the inverse step size 1/*α*, thus improving the storage efficiency of the network. Moreover, large *γ* values above 1 would guarantee a better memory capacity, even if it comes at the price of higher dispersion of the memory weights^42^. In summary, even when markedly non linear, the analysed device realizes a soft approach to the boundary, thus improving the memory performance in terms of capacity for adaptive learning under changing information input.

### Variability

**Figure 5:**
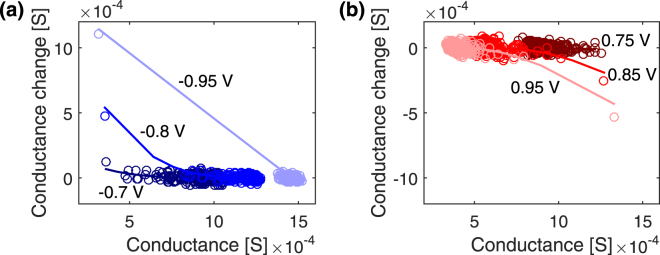
Conductance increment as a function of the conductance level. (**a**) Potentiation operations with Δ*t* = 10 *μ*s and Δ*V* = −0.7 V, −0.8 V and −0.95 V. (**b**) Depression operations with Δ*t* = 100 *μ*s and Δ*V* = 0.75 V, 0.85 V and 0.95 V.

When the device is conveniently operated, the conductance of the device can be modulated through hundreds of values (∝1/*α*). However, a fine consideration on the actually available conductance levels should take into account the overlapping variability. In order to analyse variability, the conductance change induced by each single pulse (dG) is plotted as a function of the conductance value (G) in Fig. 5 for different pulse voltages, and a fit of the renormalized soft bound law is superposed to the experimental data. Regarding potentiation (Fig. 5a), the conductance change caused by one pulse is large at low conductance values and becomes negligible and immersed in the variability as the conductance value increases. The opposite trend, together with opposite sign, is visible for depression in Fig. 5b. This evidence demonstrates the gradual approach of potentiation and depression evolutions towards the conductance boundaries. As the pulse voltage increases, dG also increases at low initial conductance value for potentiation and high initial conductance value for depression. Correspondingly, high voltage pulses (|Δ*V*| = 0.95 V) define only two conductance values, which is a clear indication of digital operation. The lower the voltage, the lower dG for each initial conductance value and the narrower the covered conductance range.

Even though the pulse series are acquired above the switching threshold, only the dG corresponding to the first pulse stands far from the x axis, while the next points follow a small trend at intermediate conductance levels which is highlighted by the soft bound law, then they accumulate along the x axis. From the scattering of dG around the x axis close to accumulation, the maximum pulse-to-pulse variability can be estimated at about 50 *μ*S. As a rule of thumb, two distinct device levels can be effectively distinguished only if their relative distance lies above the pulse variability. As an example, considering the maximum window of 1 mS obtained in the analogue region, only about 20 distinct levels can be effectively distinguished in a deterministic manner if the maximum pulse variability is considered in this device. However, it should be stressed that this is only a lower limit estimation.

Another source of variability comes from cycle repeatability. Once the device has been characterized for various programming conditions, the different switching regimes are known and any (Δ*V*, Δ*t*) couple is associated with a specific memory window and pulse dynamics. However, repeating the same pulse series numerous times will result in slightly different dynamics.

In order to probe the cycling variability, special care should be paid in choosing similar memory windows for both operations according to Fig. 3 to avoid a drift of the overall conductance for repeated potentiation and depression sequences^50^. This happens since for long enough pulse series the cumulative conductance tends to saturate to a conductance level that depends on the specific programming conditions. Moreover, as previously discussed, the device dynamics as well as the switching threshold differ between potentiation and depression operations. For this reason, in general asymmetric Δ*V* and/or Δ*t* should be applied to obtain analogue cumulative behaviours for both operations.

**Figure 6:**
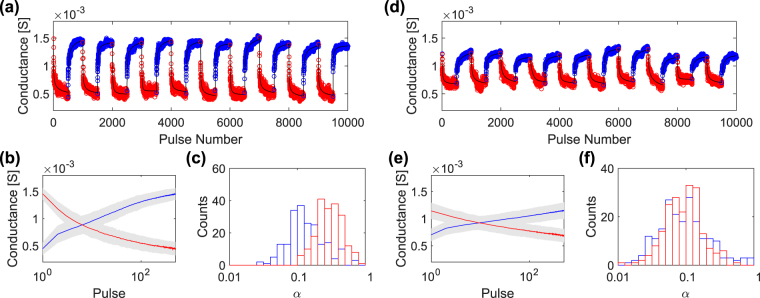
(**a**,**d**) First ten of 200 cycles programmed with 500 identical pulses of alternating depression and potentiation operations. Δ*V* and Δ*t* are +0.9 V/−0.7 V, 10 *μ*s in (**a**) and +0.75 V/−0.6 V, 100 ns in (**d**). In black line, fit to data of the generalized soft bound model. (**b**,**e**) Evolution of the average conductance (straight line) and associated standard deviation (in grey) against the number of pulses for the two data sets in (**a**,**d**). (**c**,**f**) Distributions of the *α* parameter extracted from fit.

A subset of 10 depression/potentiation over a total of 200 cycles with programming parameters chosen within the analogue region can be found in Fig. 6a. In this region, the device exhibits multiple levels with a maximum memory window of about 3. Figure 6a shows the first ten cycles programmed with Δ*t* = 10 *μ*s; Δ*V* = +0.9 V for depression and Δ*V* = −0.7 V for potentiation. 500 consecutive identical pulses were performed alternatively for each operation to ensure reaching the conductance saturation. In black line, a fit to data of the generalized soft bound law is reported (see Supplementary Fig. S4 for details). In order to better evidence the impact of cycling variability, in Fig. 6b the evolution of the average conductance as a function of the number of pulses is plotted with the associated standard deviation, in grey. A logarithmic scale is chosen for the x-axis to highlight the effect of the first pulses, which produce the greatest variation in conductance. In this plot, the dispersion appears fairly constant with an average value of 65 *μ*S, even though a decreasing trend towards the conductance boundaries can be detected (Supplementary Fig. S5).

The fit of the repeated cycles to the soft bound model allows to extract the variability of the parameters describing the conductance dynamics. The distribution of the *α* parameter, proportional to the inverse number of accessible conductance states, is reported in Fig. 6c. Quite large distribution widths of ~0.2 and ~0.3 for potentiation and depression respectively can be observed, which highlights the fairly high dispersion usually encountered in filamentary-type resistive switching devices. Nevertheless, the values of *α* clearly indicates stable and reproducible multi-state behaviour. Notably, a clear asymmetry in terms of analogue levels can be identified between depression and potentiation in Fig. 6c. This unbalance is commonly encountered for analogue switching in various resistive switching devices and has been reported previously for different systems and programming conditions^51,52^. The reason lies in the narrower range of Δ*V* at equal Δ*t* available for analogue potentiation with respect to depression for a given memory window. Indeed, it is possible to achieve symmetric operations, as visualized in the second example reported in Fig. 6d and highlighted by the *α* distribution in Fig. 6f, but a compromise has to be paid in terms of memory window. The standard deviation around the average conductance trend exhibits a mean value of about 65 *μ*S, comparable to the one detected in the previous case, as shown in Fig. 6e. However, the reduced memory window increases the incidence of conductance variation from 6% to 15% of the overall conductance span.

## Discussion

In summary, we explored the switching dynamics of HfO_2_-based resistive memory cells by sequences of identical programming pulses with the aim of enhancing the comprehension of analogue operation for improving the storage capacity in hardware neural networks. Based on the salient features of the cumulative conductance series, a fitting model was applied to provide a quantification of the degree of analogue modulation as a function of the programming pulse parameters Δ*t* and Δ*V*.

As a first observation, three main regions can be identified according to the memory window and the number of analogue states. One threshold can be defined that separates the region in which the programming pulses produce no appreciable conductance change (no switching region) from the region of analogue modulation. At higher Δ*t* or Δ*V*, a second threshold separates the analogue region from the region of digital switching. This analysis allows to estimate the programming extent of the region of analogue modulation and its dependence on the pulse parameters. The voltage width of the analogue region is about 200 mV in potentiation and slightly wider in depression. This extent is an important factor to be considered for real applications and needs to be optimized to ensure a robust analogue programming.

It is worth pointing out that also the retention of the conductance states of the devices is a critical point for the realization of networks able to learn and to record the training process and is a matter of investigation of the very recent literature^53,54^. Representative results attesting the retention at short time-scales of the present device are reported in the Supplementary Fig. S6. The long-term retention of the boundary conductance levels up to 10 years at elevated temperatures has been demonstrated in similar devices by the same authors in previous works^15,16^.

A conductance dynamics exists in the analogue region since multiple pulses are necessary to produce the maximum conductance span for the specific programming parameters, as opposed to the digital region. If the switching time is defined as the pulse Δ*t* necessary to achieve the maximum conductance span for a given Δ*V*, applying shorter pulses allows to sample different device states producing a cumulative conductance behaviour. However, the highest number of multi-state conductance levels is observed for the shortest pulses close to the switching threshold, where the memory window is also the lowest. This leads to an unavoidable compromise between number of analogue levels and programming window. This constraint is not unique to the investigated device and can be generalized to many memristive systems. In fact, even if not explicitly quantified, the same inverse relation between degree of analogue modulation and memory window can be identified in a lower current regime^23^ and in other resistive switching devices based on different materials^19,55^ and in phase change materials due to unavoidable constraints produced by the non-linear switching kinetics typical of memristive systems^46,56^.

An indication of the non linear switching kinetics in the inspected device can be identified from the voltage – time dependence of the thresholds separating the switching regimes. Indeed, an exponential dependence is identified between the voltage amplitude and the time width of the applied pulses with a slope of about 50 mV/dec in potentiation and 130 mV/dec in depression that follows previous estimations on similar material systems^46,48^. The identification for both processes of a single time–voltage slope covering almost 4 orders of magnitude of Δ*t* is the indication of a single process prevailing in the time range covered by the experiments. In other systems, *e*.*g*. in electrochemical metallization cells, two or even three distinct slopes were observed due to different limiting processes prevailing in different time–voltage regions^46^. The presence of only one time–voltage slope indicates that, throughout the entire investigated time range, the limiting process is always the same and it is usually identified with the ionic motion inside the dielectric materials which requires high local electric fields and temperatures^30,46,57^. Despite a large literature exists which deals with the switching kinetics of resistive memory devices^31,45–47,57–61^, the relation between kinetics and analog dynamics is not analysed in details. Such relation is investigated in the present paper and it allows elevating all the discussion to a level of generality that goes beyond the secondary factors influencing the switching process, e.g. space charge layers, mobility or chemical inhomogeneities (due to local and nanoscale deviations from stoichiometry and uniform ion concentration)^46,62,63^.

The asymmetric kinetics observed for the potentiation and depression processes can be explained if thermal and electro-chemical effects are considered in the system^31,51^. It was previously reported that a positive feedback loop establishes during potentiation due to a significant joule heating which accelerates the switching behaviour, leading to the steep time–voltage slope and thus to the high voltage dependence identified for potentiation^64^. At the opposite, a negative feedback loop during depression gives rise to the more gradual switching behaviour^65^. The different switching kinetics reflects also on the higher programming parameters necessary for depression with respect to potentiation to achieve the same memory window. Based on the analysis summarized in Fig. 4, the programming space available for symmetric analogue operations can be obtained by intersecting the analogue regions of the two processes while also considering similar memory windows to avoid one operation to be overwhelming with respect to the other. While a device asymmetry may not be a big concern by itself since it can be translated into an unbalance of probabilities for synapses being potentiated or depressed, the different time–voltage dependence of the analogue region restricts the space of available symmetric programming and can influence the network performance^66^.

A second major observation in the inspected conductance dynamics is the occurrence of a bounded maximum conductance span dependent on the specific programming pulses. This behaviour occurs naturally in a physical device since the maximum memory window is also limited by the switching kinetics of the system as discussed above. The main consequence is that the spacing between levels tends to diminish as the conductance limits are approached. The presence of boundaries for the maximum synaptic strength was discussed theoretically in the literature in the framework of adaptive networks^41^ and the introduction of a simple cut-off (hard bound) which sets a sudden flattening of the cumulative conductance was ruled out as optimal choice for synaptic behaviour^42^, while a gradual approach to the conductance boundaries as the one observed in the inspected device can maximize the memory capacity of the network.

Besides theoretical considerations, an approximation of evenly spaced levels, or equivalently a linear cumulative behaviour, can be obtained only in a restricted pulse interval far from conductance saturation. In a practical hardware implementation this would require additional circuitry to limit the conductance update, complicating the design of the network. On the other hand, recent works focus on material stack optimization to improve the linearity of conductance change as a benchmark for good synaptic characteristics^36,67^. This in practice corresponds to increase the number of analogue levels so that conductance saturation is pushed at higher pulse number and for practical applications the synaptic weight can be considered almost linear in the initial part of the cumulative curve. In this respect, based on the proposed analysis, the figure of merit is 1/*α*, proportional to the inverse number of levels. Considering as a benchmark the recognition of MNIST handwritten digits, Querlioz *et al*.^37^ demonstrated by network simulations that the recognition rate of the digits remains almost unvaried up to $\alpha \simeq 0.05$, then it declines rapidly for higher values, meaning that at least dozens of separate levels are necessary for this specific task. Even if the exact number of levels also depends on the specific update rule, the magnitude of this parameter can serve as a benchmark to compare different devices and different programming conditions.

Finally, for a full account of the analogue programming space, it is necessary to include also the superposed device variability since two levels may not be effectively distinguishable if the variability is greater than the level separation. Several works in the literature demonstrate the high robustness of neural network implementations to variability^37,66^. However, a gap exists between network simulations and device characterization. In this work, two types of device variability which could influence the conductance dynamics are scrutinized: pulse-to-pulse and cycling variability. Both types of variability affect the effective number of conductance levels covered by the device depending on the memory window of operation and must be taken into account to evaluate the performances of the device. In summary, the device characterization and analysis proposed in this work allow to systematically quantify the degree of analogue operation in different regimes and serves as a starting point for a reasoned evaluation of the device performance and, secondly, for the identification of specific programming conditions for practical applications. Moreover, the present work may serve as a reference for the future optimization of synaptic devices, e.g. displaying at the same time low values of the *α* parameters for both potentiation and depression, good symmetry and large conductance windows. It must be cited that only very recent works deal with the optimization of some of these features, through the use of alternative materials or combination of materials and possibly moving towards an interface dominated switching process^35,52,53,68^.

## Conclusions

In this work, we analyse the analogue conductance dynamics of TiN/HfO_2_/Ti/TiN memristive devices based on a filamentary switching mechanism. The main features of the experimental cumulative conductance change, namely the presence of conductance saturation and a slow approach to the conductance boundaries, can be reproduced by a soft bound model, which was recognized in literature as a way to improve memory capacity in the network in presence of bounded synapses. The application of this simple phenomenological model allows to clearly identify three regions of operation: no switching, gradual analog modulation and digital (almost two-level) switching as a function of the programming pulse parameters. It is found that both the demarcation lines separating the switching regimes and the memory window follow a trend in terms of pulse amplitude – timewidth that can be related to the physics of the switching kinetics in memristive devices. From this observation stems the necessary compromise between degree of analog modulation and memory window. Moreover, the different kinetics for the processes of conductance increase and decrease explain the asymmetry in terms of analogue programming space between the two operations. Finally, pulse variation and cycling repeatability are evaluated to quantify the variability superposed to the average conductance variation affecting the effective number of conductance levels. The results highlight the possibility to operate the device with robust analogue modulation and symmetric conductance processes. We develop an approach that takes into account the salient features of the device and, based on the device physics, can determine the device performance and the best operating regions and trade-offs. This analysis is fundamental for practical device implementation in hardware neural networks.

## Methods

### Fabrication of the RRAM devices

The resistance switching devices consist of a 40 nm TiN bottom electrode deposited by sputtering in a mixed Ar and N_2_ environment; 5 nm HfO_2_ switching layer deposited by atomic layer deposition (ALD) at 300 °C (Savannah 200 reactor, Cambridge) employing MeCp_2_HfMe(OMe)Hf (HfD-04) as Hf precursor and H_2_O as oxygen source; a top electrode composed of 10 nm Ti and 40 nm TiN deposited by sputtering using only Ar or mixed Ar and N_2_ environment, respectively. Due to partial oxidation of the TiN bottom electrode prior to ALD deposition^69^ and partial oxidation of the Ti top contact as a result of oxygen gettering activity, the device stack results quite symmetric and no preferential polarity exists for bipolar resistance switching operations.

### Electrical characterization

Electrical characterization was performed using a device parameter analyzer (B1500A, Keysight Technologies) equipped with high resolution source measurement units (HR-SMU) and a semiconductor pulse generator unit (SPGU, B1525A from Keysight Technologies). During DC operation, the potential was applied to the top electrode through a SMU unit, while the bottom electrode was held grounded through a second SMU unit. For pulse measurements, the SPGU unit was connected to the top electrode, while the bottom electrode was grounded through a SMU unit which provided current monitoring. An external custom switch board was built to provide a convenient selection of one of the two measurement configurations (DC or pulsed characterization)^26^.

### Device initialization

For bipolar switching operations, the devices require an initial electroforming operation. This step was carried out with a current-controlled sweep up to 300 *μ*A in order to limit the maximum current passing through the device. After the initial forming step, a few DC cycles were performed by voltage sweeps between −1 V and +1 V with 1 mA maximum current limitation during potentiation and no maximum current limitation during depression operations (Supplementary Fig. S7).

DC operations were applied to provide two well defined initial states within the resistance distributions of the high resistance state (HRS) and low resistance state (LRS). In case that the cell state lies outside the HRS or LRS resistance levels, a few DC cycles are sufficient to bring the device resistance within one of these resistance levels. Moreover, DC characteristics provide an indication of the final conductance achieved for a given maximum applied voltage. I-V curves and resistance distributions can be found in the Supplementary Fig. S7.

### Pulsed characterization

Starting from the initial state defined by DC operations within the resistance distribution of either the LRS or HRS, a pulsed characterization was carried out with a sequence of 300 identical pulses with a period of 80 ms and a reading operation at 100 mV after each pulse. Potentiation curves were obtained starting from the HRS, while depression curves were started from the LRS. In order to evaluate the effect of the programming conditions, i.e. Δ*t* and Δ*V*, after each sequence the cell was re-initialized by DC operations and a new sequence was performed with slightly modified programming parameters. Δ*V* of the applied pulses was span between 0.3 (−0.3 V) and +1 V (−1 V) with 50 mV step for depression (potentiation) operations. With the intention to avoid any irreversible damage to the memory cell, the maximum applied voltage was limited to the maximum value applied during comparatively slow DC operations. Δ*t* was varied between 100 ns and 300 *μ*s with two series per decade for both operations with a fixed pulse rise and fall time of 40 ns. It is worth to emphasize that no external current limitation was applied during pulsed characterization.

### Fitting procedure

Conductance series were fitted according to the generalized soft bound model. Even if equations (1) and (2) represent discrete recursive sequences, in first approximation the elemental step *δw*/*δn* can be integrated over *δn* to derive equations (3) and (4) for the cumulative evolution of the weight *w*, which is the quantity actually measured, as a function of the number of pulses *n*.

3 $${w}_{+}=1-{[1+\alpha (\gamma -1)n]}^{\frac{1}{1-\gamma }}$$

4 $${w}_{-}={[1+\alpha (\gamma -1)n]}^{\frac{1}{1-\gamma }}$$

Equations (3) and (4) need then to be renormalized between *G*_min_ and *G*_max_, which set the initial conductance value after DC initialization and the maximum (or minimum) saturation value as extracted from fitting. During fitting procedure, *α* was initialized close to lowest expected value (1 × 10^−3^) and the fit was let increasing this value up to 1, while *γ* was initialized close to 1 and its value was free to increase up to 10. A robust linear least-squares fitting method with least absolute residual method was applied to avoid excessive dependence on outliers given the not negligible pulse-to-pulse variability. In order to rescale the fitting equation to the actual conductance interval, the initial conductance at *n* = 0 was fixed from the pulse series, while the maximum (or minimum) conductance at the end of the pulse series was free to adjust from fit up to double of the value measured at the end of the pulse sequence (*n* = 300).

### Data availability

The datasets generated during and/or analysed during the current study are available from the corresponding author on reasonable request.

## Electronic supplementary material

Supplementary Information

## References

[1] Mead C (1990). Neuromorphic electronic systems. Proc. IEEE.

[2] Taha, T. M., Hasan, R., Yakopcic, C. & McLean, M. R. Exploring the design space of specialized multicore neural processors. In *The 2013 International Joint Conference on Neural Networks* (*IJCNN*), 1–8, 10.1109/IJCNN.2013.6707074 (2013).

[3] Indiveri G (2011). Neuromorphic silicon neuron circuits. Front. Neurosci..

[4] Mitra S, Fusi S, Indiveri G (2009). Real-time classification of complex patterns using spike-based learning in neuromorphic VLSI. IEEE Transactions on Biomed. Circuits Syst..

[5] Maschenko AA (2013). On the feasibility to apply a neural network processor for analyzing a gas response of a multisensor microarray. Sensors Actuators A: Phys..

[6] Bi G-q, Poo M-m (1998). Synaptic modifications in cultured hippocampal neurons: Dependence on spike timing, synaptic strength, and postsynaptic cell type. J. Neurosci..

[7] Indiveri G, Chicca E, Douglas R (2006). A VLSI array of low-power spiking neurons and bistable synapses with spike-timing dependent plasticity. IEEE Transactions on Neural Networks.

[8] Indiveri G, Linares-Barranco B, Legenstein R, Deligeorgis G, Prodromakis T (2013). Integration of nanoscale memristor synapses in neuromorphic computing architectures. Nanotechnol..

[9] Jeong DS, Kim KM, Kim S, Choi BJ, Hwang CS (2016). Memristors for energy-efficient new computing paradigms. Adv. Electron. Mater..

[10] Chen, H.-Y. *et al*. Resistive random access memory (RRAM) technology: From material, device, selector, 3D integration to bottom-up fabrication. *J*. *Electroceramics* **39**, 21–38, 10.1007/s10832-017-0095-9 (2017).

[11] Wang Z (2017). Nanoionics-enabled memristive devices: Strategies and materials for neuromorphic applications. Adv. Electron. Mater..

[12] Yu S, Wu Y, Jeyasingh R, Kuzum D, Wong HSP (2011). An electronic synapse device based on metal oxide resistive switching memory for neuromorphic computation. IEEE Transactions on Electron Devices.

[13] Kuzum D, Yu S, Wong H-SP (2013). Synaptic electronics: materials, devices and applications. Nanotechnol..

[14] Frascaroli J (2015). Resistive switching in high-density nanodevices fabricated by block copolymer self-assembly. ACS Nano.

[15] Frascaroli J, Volpe FG, Brivio S, Spiga S (2015). Effect of Al doping on the retention behavior of HfO_2_ resistive switching memories. Microelectron. Eng..

[16] Brivio S, Frascaroli J, Spiga S (2017). Role of al doping in the filament disruption in HfO_2_ resistance switches. Nanotechnol..

[17] Zidan, M. A., Chen, A., Indiveri, G. & Lu, W. D. Memristive computing devices and applications. *J*. *Electroceramics* 1–17, 10.1007/s10832-017-0103-0 (2017).

[18] Prezioso M (2015). Training and operation of an integrated neuromorphic network based on metal-oxide memristors. Nat..

[19] Serb A (2016). Unsupervised learning in probabilistic neural networks with multi-state metal-oxide memristive synapses. Nat. Commun..

[20] Park S (2015). Electronic system with memristive synapses for pattern recognition. Sci. Reports.

[21] Ambrogio S (2016). Neuromorphic learning and recognition with one-transistor-one-resistor synapses and bistable metal oxide RRAM. IEEE Transactions on Electron Devices.

[22] Gao B (2016). Metal oxide resistive random access memory based synaptic devices for brain-inspired computing. Jpn. J. Appl. Phys..

[23] Yao P (2017). Face classification using electronic synapses. Nat. Commun..

[24] Chu M (2015). Neuromorphic hardware system for visual pattern recognition with memristor array and CMOS neuron. IEEE Transactions on Ind. Electron..

[25] Covi E (2016). Analog memristive synapse in spiking networks implementing unsupervised learning. Front. Neurosci..

[26] Covi E, Brivio S, Frascaroli J, Fanciulli M, Spiga S (2017). Analog HfO_2_-RRAM switches for neural networks. ECS Transactions.

[27] Choi S, Shin JH, Lee J, Sheridan P, Lu WD (2017). Experimental demonstration of feature extraction and dimensionality reduction using memristor networks. Nano Lett..

[28] Covi E, Brivio S, Fanciulli M, Spiga S (2015). Synaptic potentiation and depression in Al:HfO_2_-based memristor. Microelectron. Eng..

[29] Benna MK, Fusi S (2016). Computational principles of synaptic memory consolidation. Nat. Neurosci..

[30] Waser R, Aono M (2007). Nanoionics-based resistive switching memories. Nat. Mater..

[31] Menzel S (2011). Origin of the ultra-nonlinear switching kinetics in oxide-based resistive switches. Adv. Funct. Mater..

[32] Gaba S, Sheridan P, Zhou J, Choi S, Lu W (2013). Stochastic memristive devices for computing and neuromorphic applications. Nanoscale.

[33] Chen PY, Gao L, Yu S (2016). Design of resistive synaptic array for implementing on-chip sparse learning. IEEE Transactions on Multi-Scale Comput. Syst..

[34] Chang CC (2017). Mitigating asymmetric nonlinear weight update effects in hardware neural network based on analog resistive synapse. IEEE J. on Emerg. Sel. Top. Circuits Syst..

[35] Wang Z (2016). Engineering incremental resistive switching in TaO_x_ based memristors for brain-inspired computing. Nanoscale.

[36] Woo J (2016). Improved synaptic behavior under identical pulses using AlO_x_/HfO_2_ bilayer RRAM array for neuromorphic systems. IEEE Electron Device Lett..

[37] Querlioz D, Bichler O, Dollfus P, Gamrat C (2013). Immunity to device variations in a spiking neural network with memristive nanodevices. IEEE Transactions on Nanotechnol..

[38] Chen, P. Y. *et al*. Mitigating effects of non-ideal synaptic device characteristics for on-chip learning. In *2015 IEEE*/*ACM International Conference on Computer*-*Aided Design* (*ICCAD*), 194–199, 10.1109/ICCAD.2015.7372570 (2015).

[39] Querlioz D, Bichler O, Vincent AF, Gamrat C (2015). Bioinspired programming of memory devices for implementing an inference engine. Proc. IEEE.

[40] Ziegler M, Riggert C, Hansen M, Bartsch T, Kohlstedt H (2015). Memristive Hebbian plasticity model: Device requirements for the emulation of Hebbian plasticity based on memristive devices. IEEE Transactions on Biomed. Circuits Syst..

[41] Parisi G (1986). A memory which forgets. J. Phys. A: Math. Gen..

[42] Fusi S, Abbott LF (2007). Limits on the memory storage capacity of bounded synapses. Nat. Neurosci..

[43] Brivio, S., Tallarida, G., Cianci, E. & Spiga, S. Formation and disruption of conductive filaments in a HfO_2_/TiN structure. *Nanotechnol*. **25**, 385705, http://iopscience.iop.org/0957-4484/25/38/385705/media, 10.1088/0957-4484/25/38/385705 (2014). 10.1088/0957-4484/25/38/385705 25181606

[44] Brivio, S. *et al*. Gradual set dynamics in HfO_2_-based memristor driven by sub-threshold voltage pulses. In *Proceedings of IEEE International Conference on Memristive Systems* (*MEMRISYS*), 1–2, 10.1109/MEMRISYS.2015.7378383 (2015).

[45] Huang P (2014). Analysis of the voltage-time dilemma of metal oxide-based RRAM and solution exploration of high speed and low voltage AC switching. IEEE Transactions on Nanotechnol..

[46] Menzel S, Böttger U, Wimmer M, Salinga M (2015). Physics of the switching kinetics in resistive memories. Adv. Funct. Mater..

[47] Marchewka A (2016). Nanoionic resistive switching memories: On the physical nature of the dynamic reset process. Adv. Electron. Mater..

[48] Wang, C. *et al*. Ultrafast RESET analysis of HfO_*x*_-based RRAM by sub-nanosecond pulses. *Adv*. *Electron*. *Mater*. 1700263, 10.1002/aelm.201700263, 1700263 (2017).

[49] Schönhals, A. *et al*. Role of the Electrode Material on the RESET Limitation in Oxide ReRAM Devices. *Adv*. *Electron*. *Mater*. **4**, 1–11, http://onlinelibrary.wiley.com/doi/10.1002/aelm.201700243/abstract, 10.1002/aelm.201700243 (2018).

[50] Serb A, Khiat A, Prodromakis T (2015). An RRAM biasing parameter optimizer. IEEE Transactions on Electron Devices.

[51] Brivio S (2016). Experimental study of gradual/abrupt dynamics of HfO_2_-based memristive devices. Appl. Phys. Lett..

[52] Lee D, Moon K, Park J, Park S, Hwang H (2015). Trade-off between number of conductance states and variability of conductance change in Pr_0.7_Ca_0.3_MnO_3_-based synapse device. Appl. Phys. Lett..

[53] Stathopoulos S (2017). Unsupervised learning in probabilistic neural networks with multi-state metal-oxide memristive synapses. Sci. Reports.

[54] Zhao, M. *et al*. Investigation of statistical retention of filamentary analog RRAM for neuromophic computing. In *2017 IEEE International Electron Devices Meeting* (*IEDM*), 39.4.1–39.4.4, 10.1109/IEDM.2017.8268522 (2017).

[55] Alibart F, Zamanidoost E, Strukov DB (2013). Pattern classification by memristive crossbar circuits using *ex situ* and *in situ* training. Nat. Commun..

[56] Kuzum D, Jeyasingh RGD, Lee B, Wong H-SP (2012). Nanoelectronic programmable synapses based on phase change materials for brain-inspired computing. Nano Lett..

[57] Waser, R., Dittmann, R., Staikov, G. & Szot, K. Redox-Based Resistive Switching Memories - Nanoionic Mechanisms, Prospects, and Challenges. *Adv*. *Mater*. **21**, 2632–2663, http://onlinelibrary.wiley.com/doi/10.1002/adma.200900375/abstract, 10.1002/adma.200900375 (2009). 10.1002/adma.200900375 36751064

[58] Messerschmitt F, Kubicek M, Schweiger S, Rupp JL (2014). Memristor kinetics and diffusion characteristics for mixed anionic-electronic SrTiO_3−*δ*_ bits: The memristor-based cottrell analysis connecting material to device performance. Adv. Funct. Mater..

[59] Yang, X., Tudosa, I., Choi, B. J., Chen, A. B. K. & Chen, I.-W. Resolving voltage-time dilemma using an atomic-scale lever of subpicosecond electron-phonon interaction. *Nano Lett*. **14**, 5058–5067, 10.1021/nl501710r, PMID: 25102402 (2014). 10.1021/nl501710r 25102402

[60] Luo WC (2013). Statistical model and rapid prediction of rram set speed-disturb dilemma. IEEE Transactions on Electron Devices.

[61] Cao MG (2012). Nonlinear dependence of set time on pulse voltage caused by thermal accelerated breakdown in the Ti/HfO_2_/Pt resistive switching devices. Appl. Phys. Lett..

[62] Magyari-Köpe, B., Tendulkar, M., Park, S.-G., Lee, H. D. & Nishi, Y. Resistive switching mechanisms in random access memory devices incorporating transition metal oxides: TiO_2_, NiO and Pr_0.7_Ca_0.3_MnO_3_. *Nanotechnol*. **22**, 254029, http://stacks.iop.org/0957-4484/22/i=25/a=254029 (2011). 10.1088/0957-4484/22/25/254029 21572196

[63] Padovani A, Larcher L, Pirrotta O, Vandelli L, Bersuker G (2015). Microscopic modeling of HfO_*x*_ RRAM operations: From forming to switching. IEEE Transactions on Electron Devices.

[64] Brivio S, Spiga S (2017). Stochastic circuit breaker network model for bipolar resistance switching memories. J. Comput. Electron..

[65] Fleck K (2016). Uniting gradual and abrupt set processes in resistive switching oxides. Phys. Rev. Appl..

[66] Agarwal, S. *et al*. Resistive memory device requirements for a neural algorithm accelerator. In *2016 International Joint Conference on Neural Networks* (*IJCNN*), 929–938, 10.1109/IJCNN.2016.7727298 (2016).

[67] Jang JW, Park S, Burr GW, Hwang H, Jeong YH (2015). Optimization of conductance change in Pr_1−x_Ca_x_MnO_3_-based synaptic devices for neuromorphic systems. IEEE Electron Device Lett..

[68] Park J (2016). TiO_*x*_-based RRAM synapse with 64-levels of conductance and symmetric conductance change by adopting a hybrid pulse scheme for neuromorphic computing. IEEE Electron Device Lett..

[69] Brivio S, Frascaroli J, Spiga S (2015). Role of metal-oxide interfaces in the multiple resistance switching regimes of Pt/HfO_2_/TiN devices. Appl. Phys. Lett..

