# Supplementary material for: Evidence of soft bound behaviour in analogue memristive devices for neuromorphic computing
Source: Sci Rep. 2018 May 8;8:7178. doi: 10.1038/s41598-018-25376-x (PMC5940832; doi:10.1038/s41598-018-25376-x)
Supplement: Supplementary file 1 — Supplementary Information [file 41598_2018_25376_MOESM1_ESM.pdf]

Evidence of soft bound behaviour in analogue memristive devices for neuromorphic computing

Jacopo Frascaroli[1], Stefano Brivio[1], Erika Covi[1], and Sabina Spiga[*]

[1]Laboratorio MDM, IMM-CNR, Via C. Olivetti 2, 20864 Agrate Brianza (MB), Italy.
[*]Corresponding Author: sabina.spiga@mdm.imm.cnr.it

Supplementary Information

1 Complete selection of cumulative conductance curves

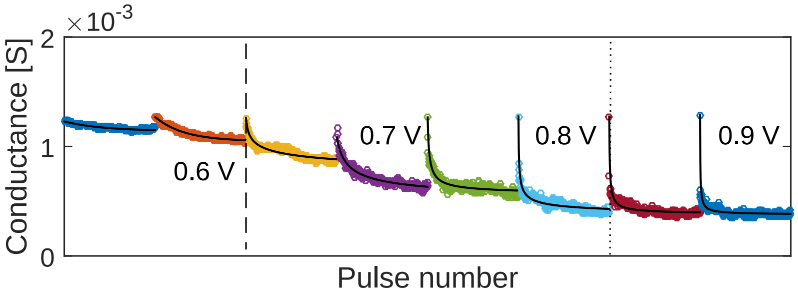

Figure S1. Conductance depression series obtained for 300 identical pulses with $\Delta t = 300$ μs. Each colour corresponds to a different series of 300 pulses starting from a similar initial state and operated with increasing absolute potential between 0.55 V and 0.9 V with a step of 50 mV.

Sequences of identical pulses produce different conductance trends depending on the programming parameters of the square pulses. In Figure S1 a series of depression operations is portrayed at fixed pulse time width of 300 μs and increasing pulse amplitude. In Figures S2a through S2d potentiation and depression curves are plotted by fixing alternatively the pulse amplitude (ΔV) or the pulse width (Δt). The variable parameter is varied by steps of 50 mV for ΔV and with two values per decade for Δt.

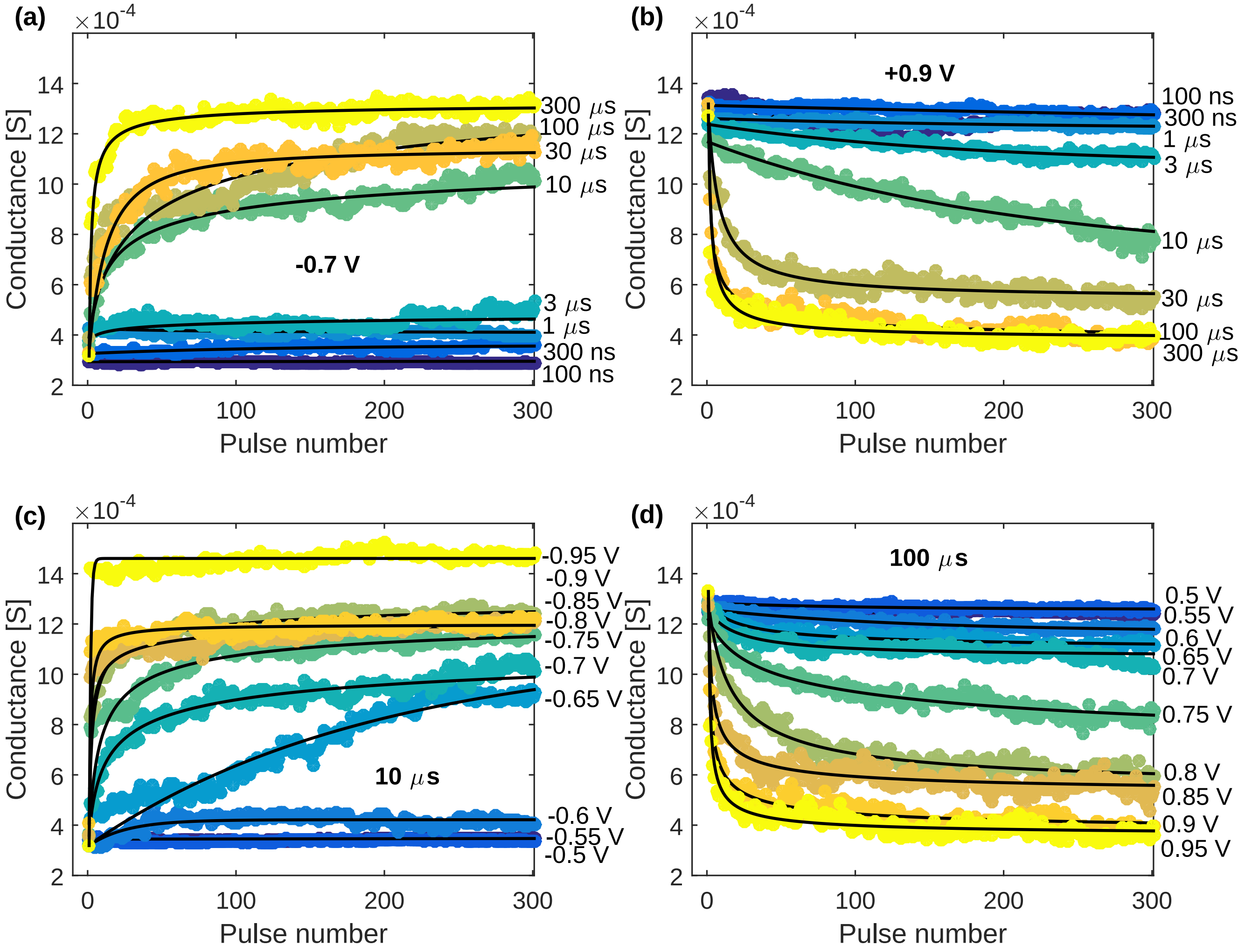

Figure S2. Sequences of 300 identical pulses for different programming conditions. In black lines, fit to data of the generalized soft bound model. (a) Potentiation operations for a fixed ΔV of -0.7 V at different Δt; (b) Depression operations operated at $\Delta V = +0.9$ V and different Δt; (c) Potentiation curves obtained at $\Delta t = 10$ μs and different ΔV; (d) Depression curves for $\Delta t = 100$ μs and different ΔV.

2 Details of the soft-bound model

In order to clarify the meaning of the α and γ parameters of the soft bound model, Fig. S3 is reported. The Figure shows the soft-bound model for potentiation for different α values and same γ (Fig. S3a) and, conversely different γ values for the same α (Fig. S3b). It can be appreciated that low α values correspond to more available conductance values. High γ values guarantee a slow approach of the boundaries but at the expenses of the effective window, even though the asymptote of all the curve in Fig. S3b is 1.

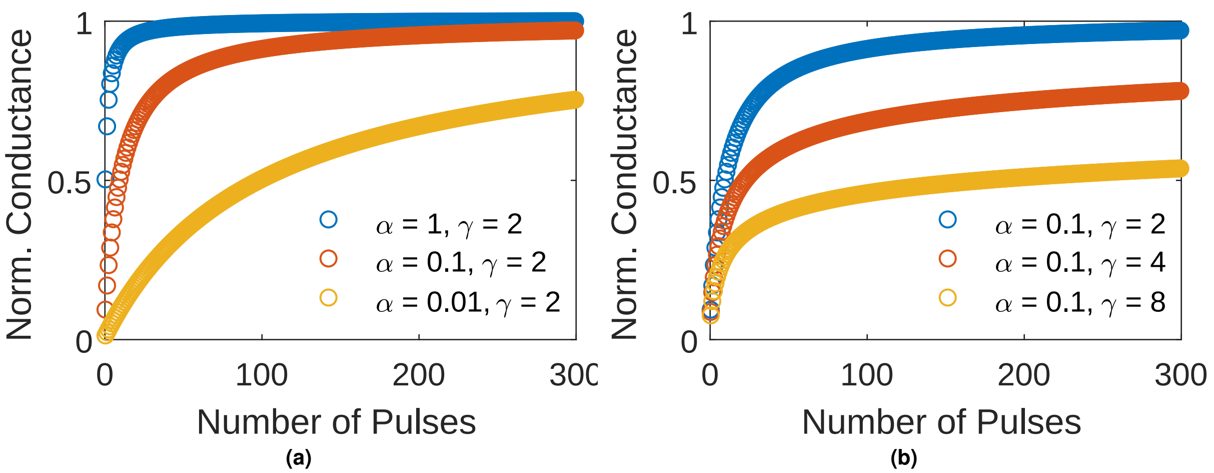

Figure S3. Soft bound model for potentiation operation for different α values and same γ (fig. S3a) and, conversely different γ values for the same α (fig. S3b).

As cited in the manuscript, a constant weight change, resulting in a linear weight dynamics, is described by a value $\gamma = 0$. A first formulation of the weight dependent update rule is the one neglecting the parameter γ (or equivalently $\gamma = 1$). Letting the parameter γ vary above unity constitute a general formulation of a weight dependent update rule.

3 Variability of repeated cycles

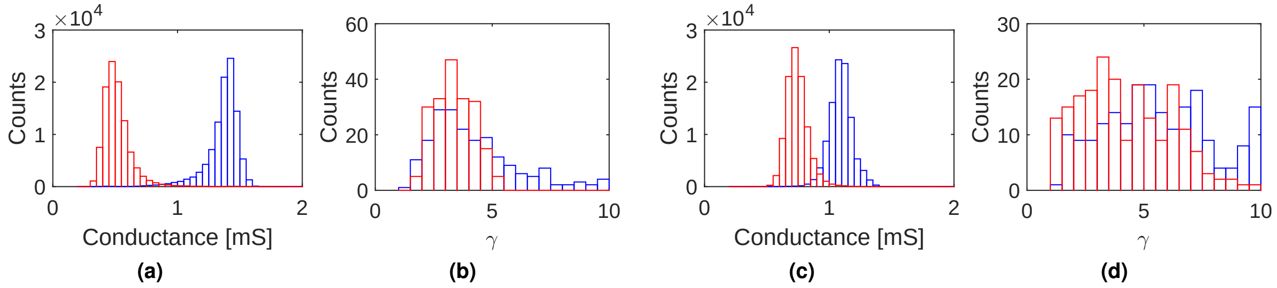

Figure S4. Histograms derived from 200 cycles programmed with 500 identical pulses of alternating depression and potentiation operations. ΔV and Δt are +0.9 V/-0.7 V, 10 μs in (a,b) and +0.75 V/-0.6 V, 100 ns in (c,d). (a,c) Histograms of the conductance values measured after each pulse for both programming conditions. (b,d) Distributions of the γ parameter extracted from fit.

Figures S4a and S4c show the distributions of the conductance values measured after each pulse for the two exemplary cycle programming conditions that appear in the paper. Blue histograms correspond to conductance potentiation, while red histograms correspond to depression operations.

The reciprocal distance between the maximum of the distributions of the conductance values can be interpreted as a measure of the dynamic range obtained for the specific programming condition. Indeed, in the cycles of Fig. S4c the memory window is reduced with respect to Fig. S4a. Additionally, the asymmetric shape of the conductance histograms towards intermediate values highlights the presence of intermediate states which are partially overlapped at the centre of the programming window. At the opposite side of the distributions, the asymmetric broadening of the distribution over the conductance extremes evidences the variability of the conductance saturation values. Higher variability of the conductance in saturation can be identified in Fig. S4c with respect to Fig. S4a, especially for the operation of conductance potentiation.

Figures S4b and S4d show the distributions of the parameters γ extracted from a fit to the generalized soft bound law for each conductance series. In the multiplicative rule for the weight update $\delta w(w) = \alpha w^{\gamma}$, the parameter γ represents the deviation from the simple soft bound behaviour and returns the dependence of the weight update from the state of the synaptic weight. As γ grows, the weight update becomes smaller. In Fig. S4b, the parameter γ is peaked between 2 and 3 for both operations consistently with values determined for the single conductance series with similar programming conditions presented in the paper. However, a significant broadening is observed due to cycle-to-cycle variations of the conductance dynamics. In the second example of Fig. S4d, the γ distributions appear even more flattened due to an enhanced cycle-to-cycle variation. However, it should be noted that in the latter programming conditions both conductance trends follow an almost exponential behaviour. Thus, the cumulative conductance trends can be well reproduced by a simple soft-bound behaviour ($\gamma = 1$) and the introduction of a second parameter in the fitting procedure appear redundant.

The standard deviation of the average conductance for 200 cycles (ΔV +0.9 V/-0.7 V; Δt 10 μs) is plotted in Fig. S5a as a function of the number of pulses. A decreasing trend can be observed from the initial pulses, which produce larger conductance variation, towards last pulses, for which the conductance has reached a saturation level and is only subject to subtle variations. It is interesting to note that for high pulse number, when the conductance is almost stable, the standard deviation approaches a level of $\sim$50 μS which is comparable to the pulse-to-pulse variability superimposed to single conductance series. In Fig. S5b the same standard deviation is replotted against the average conductance, so that the downward trend towards the conductance edges is more apparent.

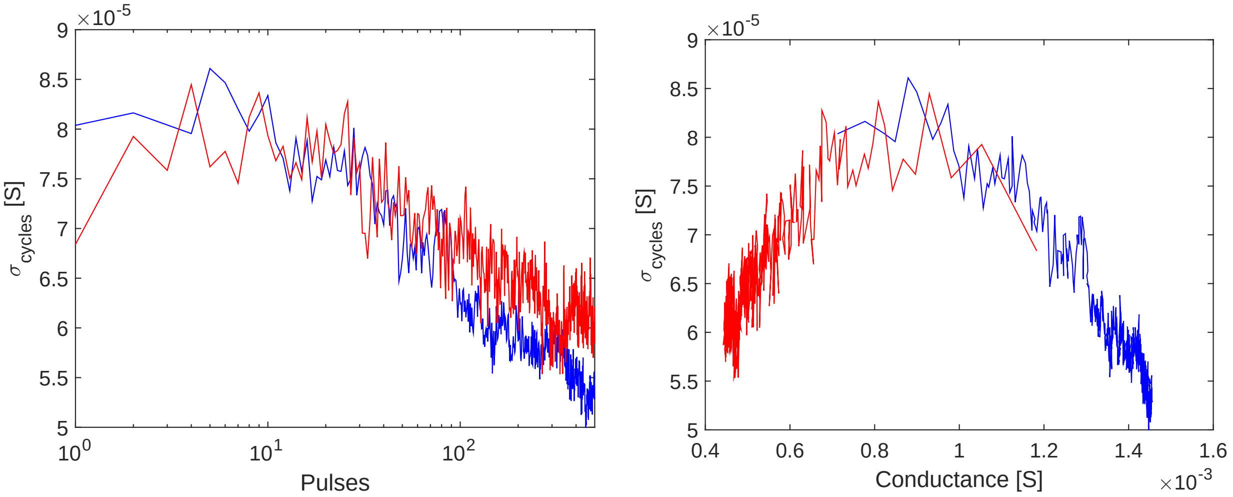

(a) **(b)**

Figure S5. Standard deviation of the average conductance determined from 200 potentiation/depression cycles (ΔV = +0.9 V/-0.7 V, Δt = 10 μs) plotted against the number of pulses (a) and against the average conductance (b).

4 State Retention

First of all, it should be mentioned that the retention of the two boundary of states filamentary devices as used for memory applications is highly reliable and extends up to 10 years at elevated temperature.[1] This result was also demonstrated by part of the same authors on similar devices.[2,3] The literature on digital memory devices also revealed that, on long time scales or elevated temperatures, the retention time depends on the resistance of the state.[4] However, considerations on the retention over long timescales (days-years) become important for evaluating the persistence of memories in an already trained network, which of course is a significant issue.

For what concerns the retention features that might influence the training procedure, one must consider the retention on short time scales. For instance, the retention on a time scale as short as few ns or μs might impact the training of high speed, high throughput neural accelerators. Conversely, the retention on the time scale of seconds-minutes can impact the training of real-time networks. It is interesting to mention an example of theoretical study (by Brader et al[5]) that considers a model of bi-stable synapse that combines short term state instability of intermediate states an long term stability of boundary states.

In this general framework, only in the very recent literature, few works deal with the retention characteristics and with short term dynamics of intermediate conductance states.[6–9] Here, an indication of the stability of the device conductance state is reported. Indeed, the short-term retention of different states, programmed through potentiation operation, is tested by monitoring the conductance evolution over time with a small non perturbing voltage of 0.1 V. The result is shown in the Fig. S6. Apart from a few glitches, all states exhibit a mostly stable resistance that surpasses 10^3 s. Moreover, in the measured time no clear distinction in retention behaviour can be observed between the resistance states.

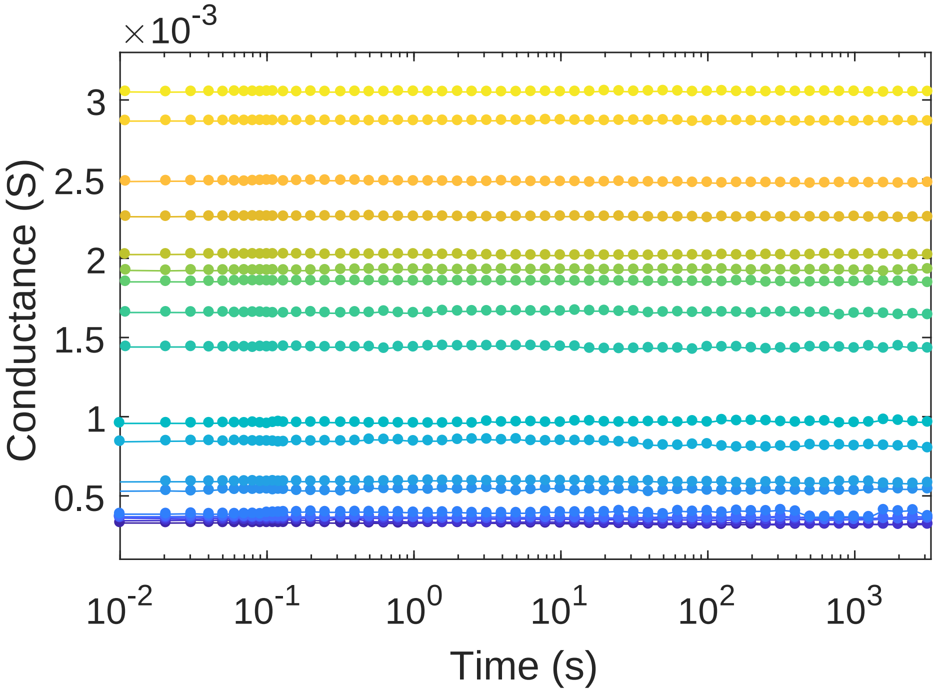

Figure S6. Retention experiment performed for different conductance states up to 10^3 s.

5 Current–Voltage curves and resistance distributions

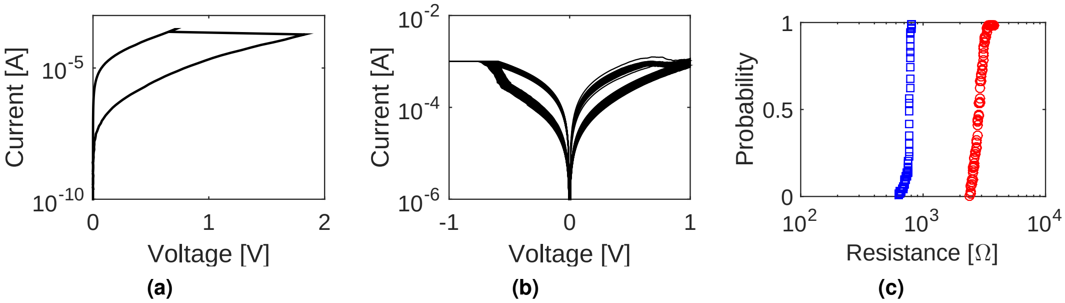

Figure S7. (a) Forming operated with a current-controlled sweep up to 300 μA; (b) plot of 300 DC cycles between -1 V and +1 V; (c) cumulative resistance distributions for the low and high resistance states obtained by DC operations.

Figure S7 displays the DC characteristics of the TiN/HfO_2/Ti/TiN RRAM device. Figures S7a and S7b show the initial forming operation operated with a current-controlled positive sweep up to 300 μA and 300 DC cycles operated by voltage sweeps between -1 V and +1 V. During the negative set processes the current compliance was set to 1 mA. A certain dispersion is shown in Fig. S7b due to repeated DC cycles. Figure S7c displays the cumulative probabilities of the low resistance state (LRS) and the high resistance state (HRS) extracted from the 300 DC curves of Fig. S7b. Two well distinguished and non overlapping distributions are found for the two states.

DC cycles were used to obtain reproducible initial states of the device prior to electrical testing by sequences of identical pulses. DC cycles were repeated until the device resistance fell within the target resistance interval. This interval was set based on the cumulative resistance distribution of Fig. S7c between 20 % and 80 % of the cumulative probability for each distribution.

References

1. International technology roadmap for semiconductors 2.0 2015 edition. URL http://www.itrs2.net/.

2. Brivio, S., Frascaroli, J. & Spiga, S. Role of Al doping in the filament disruption in HfO_2 resistance switches. *Nanotechnol.* **28**, 395202 (2017). URL http://stacks.iop.org/0957-4484/28/i=39/a=395202.

3. Frascaroli, J., Volpe, F. G., Brivio, S. & Spiga, S. Effect of Al doping on the retention behavior of HfO_2 resistive switching memories. *Microelectron. Eng.* **147**, 104–107 (2015). URL http://www.sciencedirect.com/science/article/pii/S0167931715002555. DOI 10.1016/j.mee.2015.04.043.

4. Ielmini, D. Resistive switching memories based on metal oxides: mechanisms, reliability and scaling. *Semicond. Sci. Technol.* **31**, 063002 (2016). URL http://stacks.iop.org/0268-1242/31/i=6/a=063002. DOI 10.1088/0268-1242/31/6/063002.

5. Brader, M., Senn, W. & Fusi, S. Learning Real-World Stimuli in a Neural Network with Spike-Driven Synaptic Dynamics. *Neural Comput.* **19**, 2881–2912 (2007). URL http://www.mitpressjournals.org/doi/abs/10.1162/neco.2007.19.11.2881.

6. Kim, S. *et al.* Experimental Demonstration of a Second-Order Memristor and Its Ability to Biorealistically Implement Synaptic Plasticity. *Nano Lett.* **15**, 2203–2211 (2015). URL http://dx.doi.org/10.1021/acs.nanolett.5b00697. DOI 10.1021/acs.nanolett.5b00697.

7. Du, C., Ma, W., Chang, T., Sheridan, P. & Lu, W. D. Biorealistic Implementation of Synaptic Functions with Oxide Memristors through Internal Ionic Dynamics. *Adv. Funct. Mater.* **25**, 4290–4299 (2015). URL http://onlinelibrary.wiley.com/doi/10.1002/adfm.201501427/abstract. DOI 10.1002/adfm.201501427.

8. Zhao, M. *et al.* Investigation of statistical retention of filamentary analog RRAM for neuromophic computing. In *2017 IEEE International Electron Devices Meeting (IEDM)*, 39.4.1–39.4.4 (2017). DOI 10.1109/IEDM.2017.8268522.

9. Stathopoulos, S. *et al.* Multibit memory operation of metal-oxide bi-layer memristors. *Sci. Reports* **7** (2017). URL http://www.nature.com/articles/s41598-017-17785-1. DOI 10.1038/s41598-017-17785-1.
